# Evidence for Ventilation through Collective Respiratory Movements in Giant Honeybee (*Apis dorsata*) Nests

**DOI:** 10.1371/journal.pone.0157882

**Published:** 2016-08-03

**Authors:** Gerald Kastberger, Dominique Waddoup, Frank Weihmann, Thomas Hoetzl

**Affiliations:** University Graz, Institute of Zoology, Graz, Austria; University of Cologne, GERMANY

## Abstract

The Asian giant honeybees (*Apis dorsata*) build single-comb nests in the open, which makes this species particularly susceptible to environmental strains. Long-term infrared (IR) records documented cool nest regions (CNR) at the bee curtain (n_CNR_ = 207, n_nests_ > 20) distinguished by marked negative gradients (ΔT_CNR_/d < -3°C / 5 cm) at their margins. CNRs develop and recede within minutes, predominantly at higher ambient temperatures in the early afternoon. The differential size (ΔA_CNR_) and temperature (ΔT_CNR_) values per time unit correlated mostly positively (R_AT_ > 0) displaying the Venturi effect, which evidences funnel properties of CNRs. The air flows inwards through CNRs, which is verified by the negative spatial gradient ΔT_CNR_/d, by the positive grading of T_CNR_ with T_amb_ and lastly by fanners which have directed their abdomens towards CNRs. Rare cases of R_AT_ < 0 (< 3%) document closing processes (for ΔA_CNR_/Δt < -0.4 cm^2^/s) but also suggest ventilation of the bee curtain (for ΔA_CNR_/Δt > +0.4 cm^2^/s) displaying “inhalation” and “exhalation” cycling. “Inhalation” could be boosted by bees at the inner curtain layers, which stretch their extremities against the comb enlarging the inner nest lumen and thus causing a pressure fall which drives ambient air inwards through CNR funnels. The relaxing of the formerly “activated” bees could then trigger the “exhalation” process, which brings the bee curtain, passively by gravity, close to the comb again. That way, warm, CO_2_-enriched nest-borne air is pressed outwards through the leaking mesh of the bee curtain. This ventilation hypothesis is supported by IR imaging and laser vibrometry depicting CNRs in at least four aspects as low-resistance convection funnels for maintaining thermoregulation and restoring fresh air in the nest.

## Introduction

The Asian giant honeybees (*Apis dorsata*) build their hemi-circular nests [1–13] in the open, which makes *A*. *dorsata* particularly prone to diurnal and seasonal changes in the environment (*A*. *dorsata*: [7–15], honeybees: [16–26]), particularly regarding the exposure to sun, wind and rain. The singular comb is covered on both sides with multilayers of worker bees (termed as “bee curtain” [12]), which enables brood incubation within narrow temperature limits at 35°C (*A*. *dorsata*: [13–15]; honeybees: [16–25]), despite constrictions due (a) to the choice of the nest location [5–14], (b) to the functional topology of the bee curtain [6–13], (c) to defence [5–8, 27–34] or (d) periodic mass flight activity [35–37].

For example, (a) the preference for southward nest orientation maximizes sun irradiation at one of the nest sides but leaves the other side in the shade [6–11]. (b) The performance of the bee curtain changes regularly, compacting under cooler conditions (quite similar to winter clustering of *A*. *mellifera* [21–26]) and widening up under warmer conditions (forming a wider meshed, sometimes beard-like structure; additionally, curtain bees may roam over cooler structures, e.g. alongside the upper nest parts [6–8]). (c) The nest also transforms into a looser formation in defence mode, in particular in preparation for the mass release of flying guards [5–8, 12, 34]. (d) Regularly, periodic mass flight activity [35–36] dramatically affects the interior nest milieu, when a good part of colony members set off to defecate, which is discussed to serve for dumping excessive heat [13–14, 37].

Honeybees control homoeothermy of the nest interior by heating up the flight muscles (*Apis dorsata*: [13–15, 38–39]; honeybees: [16–26, 40–45]), in particular through empty-cell heating [19–20, 46–47] and by collective cooling by evaporating a thin film of water over rims and caps of cells [16–26, 40–55] and via crop-regurgitation responses [25, 53]. Re-ingestion of droplets of water or watery honey cooled by regurgitation has also been observed in giant honeybees [13–15] whereby, as *in A*. *mellifera*, the number of bees engaged in such “gobbetting” behaviour increases with rising ambient temperature.

Individual honeybees are capable of initiating airflows, whilst remaining in a stationary position. In *A*. *mellifera* and *A*. *cerana* this behaviour [56–58] happens in front of the beehive, by fanning towards [46, 56] or away from the nest [57–58], or even inside the beehive [25] supporting the cooling by evaporation and the ventilation of the nest interior [40–56].

Long-term infrared recording of *A*. *dorsata* nests [39] brought evidence of a further, so far unknown potential for thermoregulation and ventilation in honeybees: Regularly, cool nest regions (CNRs) emerge on the bee curtain within minutes and fade away minutes or hours later (S1 Movie). We stipulate here the *funnel hypothesis* assuming that CNRs are gates through which ambient air flows into the nest interior. The further-going *ventilation hypothesis* proposes that colonies open transitory pathways such as CNR funnels to constitute and maintain the appropriate milieu inside the bee curtain, adaptive for brood incubation. Two prerequisites of CNRs are here mandatory to verify both hypotheses: first, CNRs should preferentially occur under higher ambient temperatures when the need of thermo-ventilation rises, and not incidentally without any relation to environmental conditions; and second, they should facilitate cycling airflows to establish the proper interior nest milieu regarding temperature, humidity and fresh air.

This paper substantiates both hypotheses by quantitative data, describing for the first time the functional properties of CNR funnels in *A*. *dorsata* nests regarding their occurrence, their physical properties, and their significance in fanning-supported ventilation.

### Ethics Statement

The office of the Rector of the Centre for International Relations of the Tribhuvan University (Kathmandu, Nepal) supported the research expeditions 2009 and 2010 entitled ‘‘Study on the behaviour of the giant honeybees: Observations and Recording of behaviours at the nesting site” in the Chitwan district of Nepal.

## Material and Methods

### Species and study site

**Fig 1:**
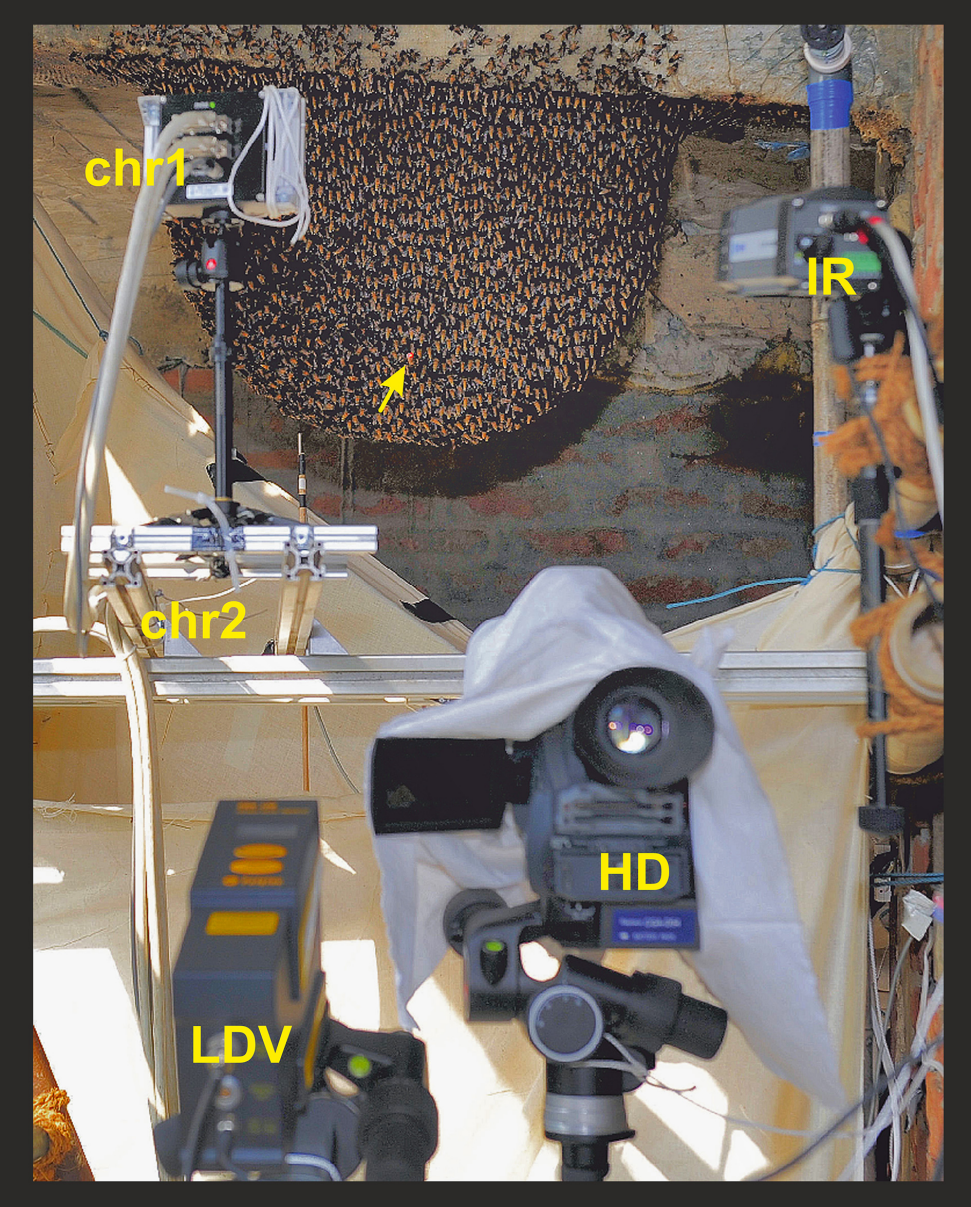
Experimental setup. The experimental *Apis dorsata* nest in a bay at the shaded rear side of the second floor of a hotel in Chitwan (Nepal); built at a traditional nesting site, which can be discerned by the wax traces from previous years. In front of the nest on the unsecured balcony the recording devices were placed: IR, infrared camera; HD, high definition camera; chr1 & chr2, two additional high resolution cameras for other purposes [28–31]; LDV, laser Doppler vibrometer [30]. The yellow arrow marks the spot of the red LDV light reflected at the end plane of a rod, which had been stuck through the comb (see also schematics in S3 Fig).

**Fig 2:**
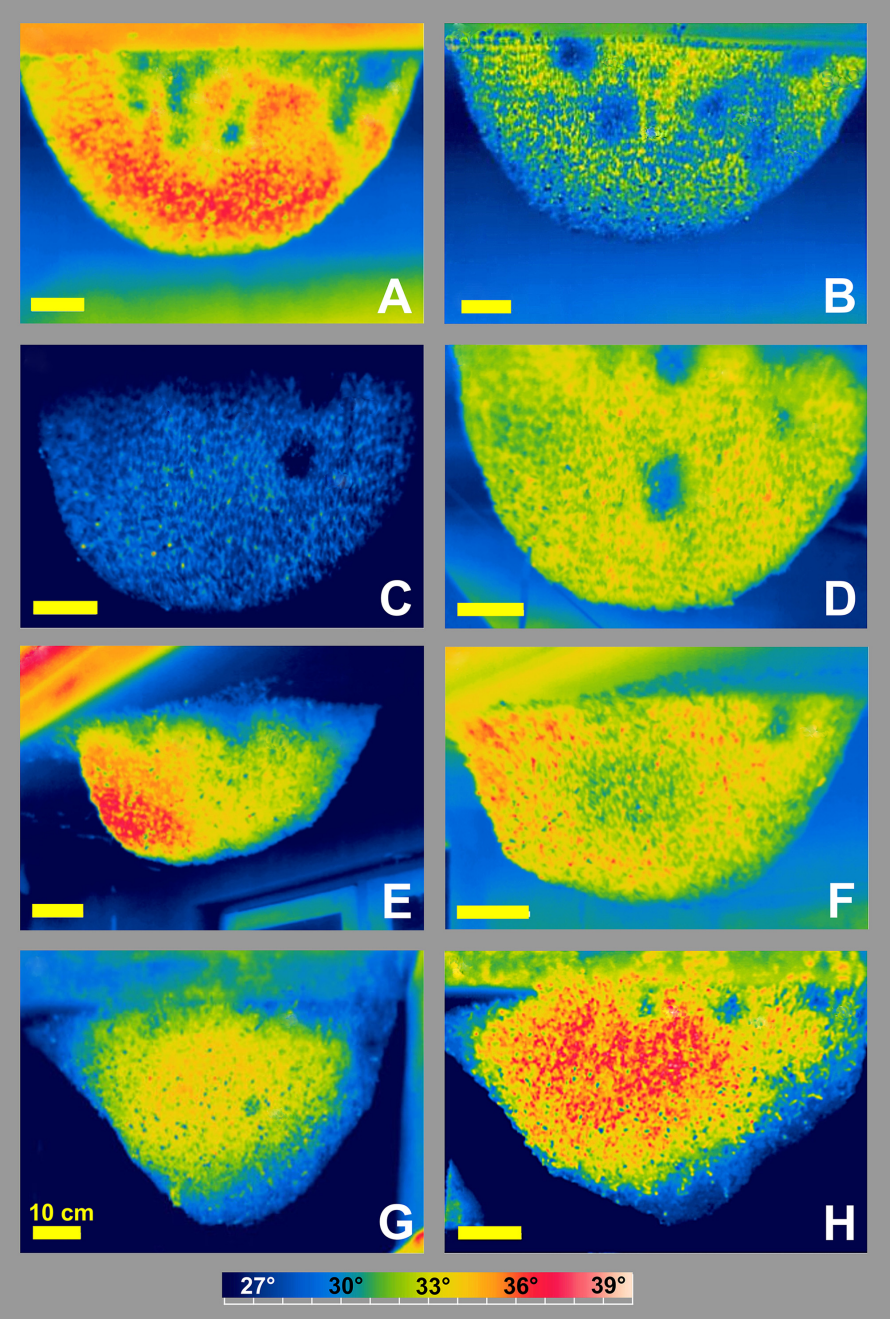
*Apis dorsata* nests (Chitwan, Nepal) with cool nest regions (CNRs). (A-B) Experimental “nest 01” recorded at 3:16 pm and 2:11 pm under strong solar irradiation revealing 6–8 CNRs; (C-D) “nest 02”, morning aspect (10:15 am) with four CNRs, and afternoon aspect (4:12 pm); (E-F) “nest 03”, exposure to direct sunlight from the left at 1:11 pm and 4:12 pm; (G-H) two days old colony cluster, late morning aspect at 11:05 am and at 1:22 pm under strong solar irradiation. Horizontal yellow bars indicate 10 cm; bottom bar, rainbow-900 temperature scale (26°- 40°C).

The main experiments were conducted in November 2010 with nine colonies of giant honeybees (*Apis dorsata dorsata* [59]) at two different sites in Sauraha near Chitwan National Park (Nepal). Additional observations refer to previous expeditions in India (1998) and Nepal (2003, 2009). The nests selected (Fig 1; S1 Fig) were attached mostly on houses to concrete overlaps and were in different development stages [1–13]. Generally, *A*. *dorsata* nests may appear, when newly arrived from migration, bivouac like without or with a small (< 1 dm^2^) central comb (Fig 2, panels G-H), but some weeks after arrival, they represent fully established nests (Fig 2, panels A-F; Tables 1 and 2) with a central comb with larvae, honey and pollen cells [1–13], covered by a multi-layered bee curtain.

**Table 1:** Benchmark properties of the bee curtain of a giant honeybee nest under normative conditions.

Pos	Parameter	Abbreviation	Value	Dimension	Reference
1	Mass of a single bee or single larva	M_bee_	140	[mg]	own experience
2	Occurrence of bees in the bee curtain	V_bee_	1	[cm^-3^]	own experience
3	Number of layers in the bee curtain of one side	N_layers_	5		own experience
4	Mass of the bee curtain on the affected (ipsilateral) side	M_bc_ipsi_	70.71	[g dm^-2^]	Pos 1–3
5	Mass of the bee curtain on the non-affected (contralateral) side	M_bc_contra_	70.71	[g dm^-2^]	Pos 1–3

**Table 2:** Benchmark properties of the comb of a giant honeybee nest under normative conditions.

Pos	Parameter	Abbreviation	Value	Dimension	Reference
1	Mass of wax of the comb	M_wax_	24.12	[g dm^-2^]	[72]
2	Mass of the larvae in the comb	M_larvae_	220.36	[g dm^-2^]	[72]
3	Mass of larvae-containing comb cells	M_comb_	134.29	[g dm^-2^]	[72]
4	Mass of the larvae-containing comb cells plus the mass of the contralateral bee curtain	M_comb_ + M_bc_contra_	205.00	[g dm^-2^]	S3 Fig
5	Number of cells at both comb sides	N_cells_	787	[dm^-2^]	[72]

### Recording and image analysis

The experimental nests (e.g. Fig 1) were filmed through 200 h of observation time from a distance of 1.5 m with a high-definition (HD) camera (Panasonic HVX 200) and in parallel, with an infrared (IR) camera (Flir A320; resolution: 640 x 560 pixel; 9 Hz). The color-coded (rainbow-900 palette) IR images were transferred from the IMG-format (providing adjustable temperature scales when operated by Flir-Researcher 2.9) into the fixed-scale BMP-format, which was further analysed by the Image-Pro software (MediaCybernetics 7.0). In total, > 30 000 frames were selected for the final evaluation representing 120 min recording time. Selected images from both HD and IR films were processed by the ThermaCAM Reporter 9.1 Professional (Flir) and Image-Pro and superposed by triangulation to the view of the IR camera. This allowed the identification of the temperature values of bee bodies and of the cavities between.

Data loggers (HOBO U12) were mounted one meter in front and behind the nest and registered ambient humidity, irradiation and ambient temperature (T_amb_), which latter was also used to calibrate the IR camera.

### Spatial and thermal properties of cooler regions on the nest surface

**Fig 3:**
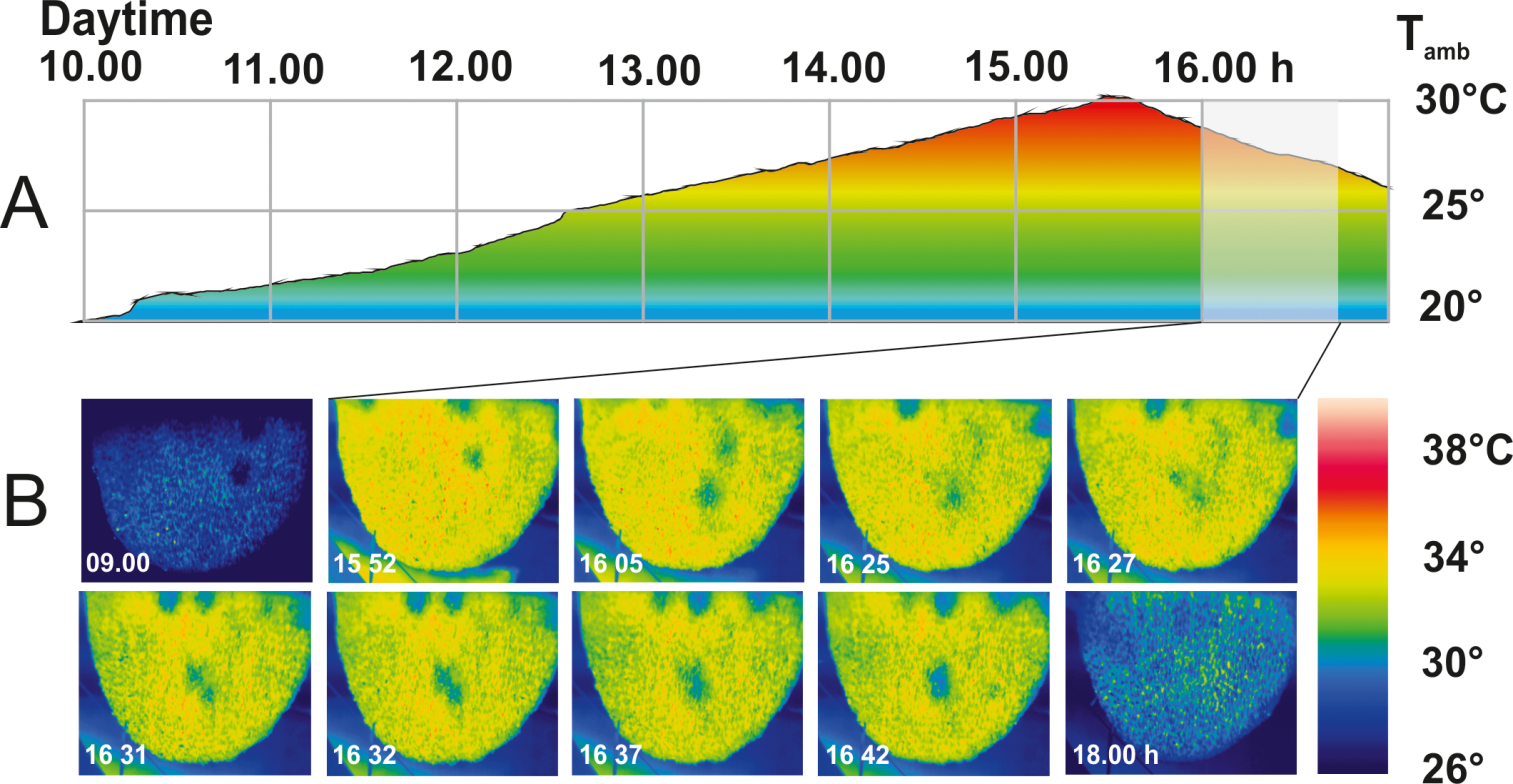
CNRs during daytime (experimental “nest 02”; 04 11 2010; Chitwan, Nepal). (A) Plot of ambient temperature T_amb_ [°C] (ordinate) versus daytime (abscissa). (B) IR images in the morning (09:00 h), at the climax of ambient temperature (15:52–16:42 h) with up to six CNRs, and in the evening (18:00 h). Temperature scale at the right side (26°- 40°C).

**Fig 4:**
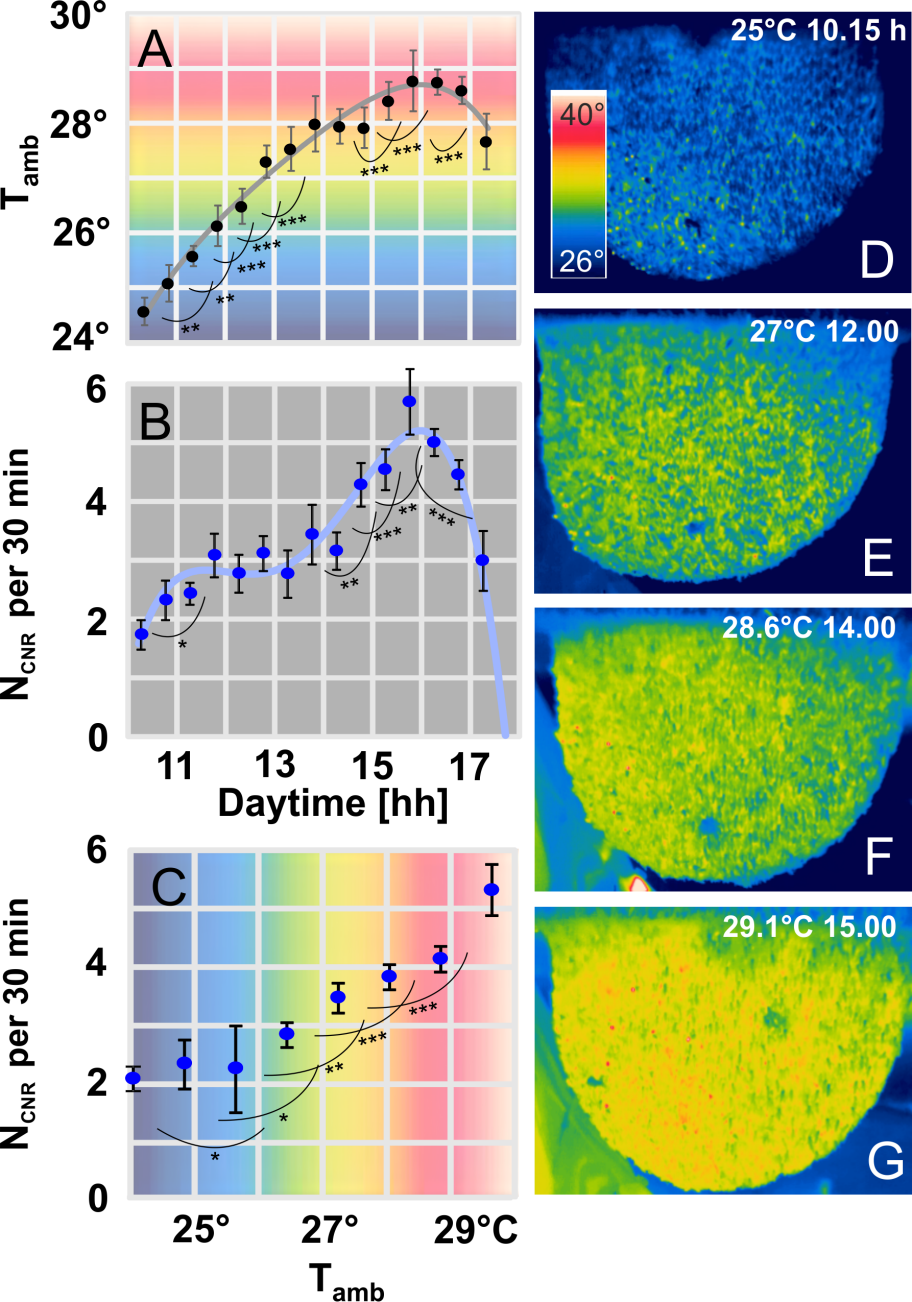
The occurrence of CNRs. (A) Plot of ambient temperature in °C (ordinate: T_amb_) over daytime in hours (abscissa). Pooled data from “nest 02” assessed in steps of 30 min (n = 209 measurements; n_exp_ = 11 experiments; Nov. 2010, Chitwan, Nepal); grey line, regression of arithmetical means: R^2^ = 0.9785. (B-C) Occurrence of CNRs (total N_CNR_ = 208) per 30 min over daytime (panel B: R^2^ = 0.9631) and T_amb_ (panel C: R^2^ = 0.9754). Stars give significant differences between adjacent pairs of data (*, P < 0.05; **, P < 0.01; ***, P < 0.001; t-test). Full circles, means; vertical bars, mean errors. The background colours symbolize the temperature gradient from cool (blue) to warm (red). (D-G) IR images at different T_amb_ and day times (see white text insets); scale bar (inset D), temperature range in °C.

**Fig 5:**
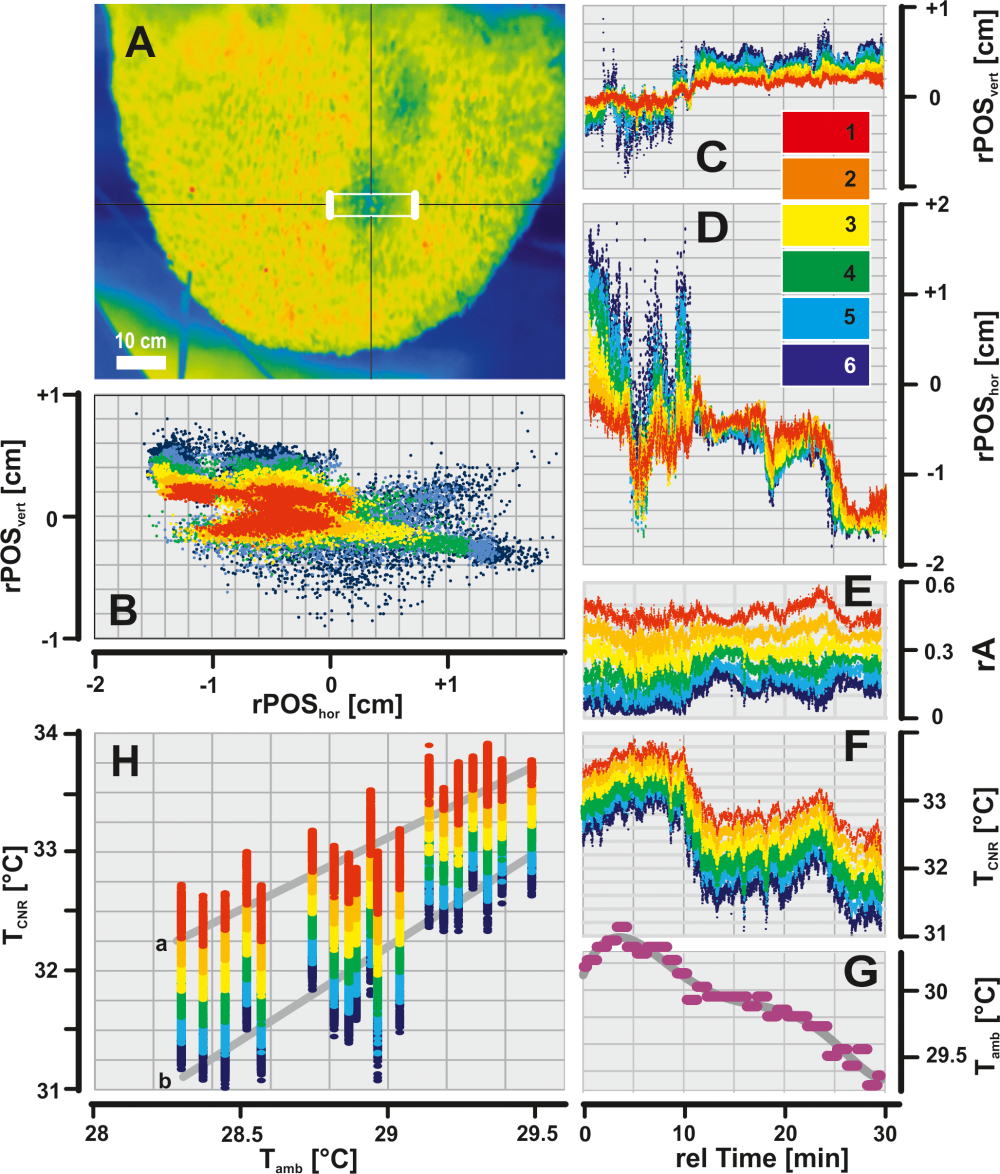
Dynamics of a CNR temperature profile. (A) IR image of the experimental “nest 02” (Chitwan, Nepal); white rectangle gives the evaluation area (A_env_), whereby the thicker side lines give the pixel positions of the frame-specific “surrounding temperature” T̃_surr_ (see Eq 3); inset scale, temperature in °C. (B) Positional dynamics of the CNR over observation time as displayed by the temperature profile with six equidistant, relative partitions (coded as red, orange, yellow, green, blue and dark blue) ranging from T_min_ to T_mid_ (see inset scale in panels C-D and Eqs 1–3); the frame-specific gravity points (n_ff_ > 7000 IR frames) of the six partitions, plotted in relative horizontal and vertical coordinates in cm; _r_POS_hor_, _r_POS_vert_: frame-specific relative positional data (cf. Eq 1) of the selected CNR (absolute values of partition 1 were set zero at the starting position, at 15:32 h); see panels C-D for the time courses of the relative _r_POS_hor_ and _r_POS_vert_ data. (E) The relative size (_r_A) of the six partitions over rel time in min; the ordinate value _r_A = 1.0 refers to the double size of A_CNR_ (see Eq 2) at the start of observation, calculated by 2 x A_CNR_ = A_env_ x 0.8 = 11.57 cm^2^; the frame wise sum of the relative sizes of the six partitions was _r_A = 1.0. (F-G) The time course of the median values T̃_CNR_ of the six partitions and of the ambient temperature (T_amb_). (H) Correlation of the singular T_CNR_ and T_amb_ values; regression functions: (a) partition 1, R^2^ = 0.7586; (b) partition 6, R^2^ = 0.7172; the T_CNR_ values grade significantly with T_amb_; e.g. for partition 1: T_CNR_ = 32.5418 ± 0.0039°C (n_IR_ = 1 635 IR images) for T_amb_ = [28.30–28.65°C], T_CNR_ = 33.6524 ± 0. 0026°C (n_IR_ = 2 112) in T_amb_ = [29.19–29.55°C]; P << 0.001 (t-test).

Giant honeybee nests occasionally display *cool nest regions* (CNRs), which are distinguished by a distinct spatial temperature gradient, and they are variable in size and position ([39], Figs 2–4; S1 Fig; S1 Movie). For evaluation, selected CNRs were enveloped by a rectangular area A_env_ (as illustrated by the white frame in Fig 5, panel A), adjusted automated or manually every 50–200 frames that the total number of pixel areas associated to the sink of CNR (n_px_sink_) was lower than 80% of the enveloped area A_env_. Subsequently, the RGB coded pixel areas of the BMP images were re-calibrated as temperature (T) values in °C by the luminance values of the associated IR scales using Image Pro software. The pixel areas (a_px_) inside A_env_ were processed horizontally (i_px_H_ = 1. n_px_H_) and vertically (i_px_V_ = 1. n_px_V_) along line profiles, with the total number of n_px_env_ [A_env_] = n_px_H_ x n_px_V_. The frame-specific position of the gravity point of the sink region (X_sink_, Y_sink_; Eq 1) regards to the positions of only those pixel areas which can be allocated up to 1°C above the minimum temperature T_min_.

For further evaluation of the sink characteristics of a CNR, the 80% of pixel areas sorted as the lower temperature part were defined as the frame-specific reference area with A_ref_ = n_px_env_ [A_env_] x 0.80. The pixel areas allocated to this lower temperature part of A_env_ were sorted along i_px_50%_ = 1 to n_px_50%_ from T_min_ (represented by the pixel area a_ipx_50%_ at i_px_50%_ = 1 with the lowest temperature) upwards to T_mid_ (represented by the pixel area a_ipx50%_, which revealed the mid temperature T_npx_50%_ at i_px_50%_ = n_px_50%_). The frame-specific temperature of the CNR was then characterized by the area A_mid_, the sum of the 50% of the lower temperature pixels a_ipx_50%_ (with i_px_50%_ = 1 to n_px_50%_) of A_ref_ (Eq 2). This algorithm was important to exclude rim-related noise in CNRs and to allocate the representative sink value of CNRs defined as T̃_sink_ (Eq 3) and as the area A_CNR_ scaled in cm^2^ (Eq 2) to the position calculated as the gravity point with the coordinates X_sink_ and Y_sink_ (Eq 1).

$${\text{X}}_{\text{sink}}=\frac{\sum_{\text{ipx}=1}^{{\text{n}}_{\text{px}\_\text{sink}}}{(\text{x}}_{\text{ipx}})}{{\text{n}}_{\text{px}\_\text{sink}}}\;{\text{and}\;\text{Y}}_{\text{sink}}=\frac{\sum_{\text{ipx}=1}^{{\text{n}}_{\text{px}\_\text{sink}}}{(\text{y}}_{\text{ipx}})}{{\text{n}}_{\text{px}\_\text{sink}}}$$ (1)

with x_ipx_ and y_ipx_ as the horizontal and vertical coordinates of those pixel areas (with 1 < i_px_ < n_px_sink_) which were sorted according to their temperature values T_ipx_ from i_px_ = 1 (with T_1_ ≡ T_min_) up to i_px_ = n_px_sink_ (with T_npx_sink_ ≡ T_min_ + 1°C).

$${\text{A}}_{\text{mid}}=\sum_{\text{ipx}=1}^{{\text{n}}_{\text{px}\_\text{50}\%}}{\text{a}}_{\text{i}}\;\text{scaled in c}{\text{m}}^{2}\;\text{according to}\;{\text{A}}_{\text{CNR}}={\text{A}}_{\text{mid}}/{\text{F}}_{\text{c}}\;\left[\text{c}{\text{m}}^{2}\right]$$ (2)

with F_c_ = 27.5625 px / cm^2^ as calibration factor for the main experiment in the BMP-formatted IR image to determine the real-world sizes of CNRs.

$${\tilde{\text{T}}}_{\text{sink}}={\text{T}}_{\text{mid}}=\text{MEDIAN}\;({\text{T}}_{\text{ipx}=1}\ldots {\text{T}}_{\text{npx}\_\text{50}\%})$$ (3)

with T_Ipx = 1_ equals T_min_ and T_np_50%_ equals the temperature of the pixel area a_npx_50%_ which is sorted at the lower half temperature part of A_ref_ according to n_px_50%_ = n_px_env_ [A_env_] * 0.80 /2.

The frame-specific relative median temperature value of the CNR (ΔT̃_CNR_) was defined according to

$$\Delta {\tilde{T}}_{CNR}={\tilde{T}}_{sink}\;\text{-}\;{\tilde{T}}_{surr}$$ (4)

with T̃_surr_ as the median of the temperature values of those pixel areas collected along surrounding lines of the enveloping rectangle of A_env_ outside the respective CNR.

These definitions allowed an automated evaluation of the temperature-dependent, positional and areal structure of CNRs over time.

### Monitoring of fanning bees

The HD documents were manually checked to confirm that the assemblage of the bee curtain remained unaltered during the emergence of CNRs and to identify fanning bees on the nest. Generally, fanning bees (Fig 3, panel A; S2 Movie) clung with their extremities to other surface bees, typically pointing away with their heads from the nest while their abdomens and thus, the air streams provoked by them were directed towards the nest. The total number of IR frames with simultaneous HD records was n_ff_ > 24 000 amounting to 76.63% images under fanning conditions (“fanners were identified at CNRs”) and to 23.38% images under non-fanning conditions (“no active fanners were identified at CNRs”).

### Laser vibrometry

A laser Doppler vibrometer (abbreviated further as “LDV”; Polytec PDV 100) was positioned on the experimental (further termed as “ipsilateral”) side of the nest (Fig 1). A wooden rod with round diameter (8 x 100 mm; 1.28 g) was stuck into the comb at a central position [30], whereby the ipsilateral end protruded slightly over the surface of the bee curtain. A piece of white paper was glued to this plane end serving as a reflector for the laser beam of the LDV. The firm connection of the rod with the comb allowed the measurement of the directional z-component of the comb movement (ΔZ_comb_/Δt) utilizing the Doppler phase shift between emission and reflection of the LDV signal. By definition, the direction of these dislocations of the comb can be discerned simply by the sign of the shifts (ΔZ_comb_/Δt < 0: „towards“, and ΔZ_comb_/Δt > 0: „ away from”the ipsilateral LDV position).

The raw LDV data with a resolution of <0.05 μm/s and sampled at intervals of 20 μs were further processed by MATLAB through a low-pass Butterworth IIR filter with a cut-off frequency of 250 Hz and displayed in data streams, frequency spectrograms and single-sided Fourier spectra. Finally, the raw Butterworth-filtered LDV-data streams were offset-corrected, weighted due to an insufficiency of a high-pass operation of LDV, by assigning an attenuation factor estimated at f_att_ = 0.667 (Table 3, Pos 2), and were lastly scaled as z-velocities in mm/s or integrated as z-dislocations in mm.

**Table 3:** Benchmark properties of hypothetical inhalation-exhalation cycling (hIEC) of a giant honeybee nest under normative conditions.

Pos	Parameter	Abbreviation	Value	Dimension	Reference
1	Mean maximal dislocation of the comb (uncorrected regarding Pos 2)	MAX ΔZ_comb_	39.05 ± 3.82	[μm]	n = 23 LDV episodes [30]
2	Attenuation factor due to the high-pass operation of LDV	f_att_	0.667		own estimate
3	Mean maximal dislocation of the comb (corrected regarding Pos 2)	MAX ΔZ_comb_	58.58 ± 5.73	[μm]	Pos 1–2
4	Mean maximal dislocation of the ipsilateral bee curtain	MAX ΔZ_bc_ipsi_	169.84	[μm]	Eq 4
5	Presumed area (radius) around CNR affected by the hIEC, taken for the mathematical model	A_ref_ (r_ref_)	1000 (17.85)	[cm^2^] ([cm])	own definition
6	Frequency of the hIEC, as supported by LDV	f_IEC_	0.0090942	[Hz]	Fig 9, panel C
7	Period of hIEC	p_IEC_	109.96 ± 0.55	[s]	Frequency band: 0.0025–0.1 Hz Fig 9, panel C
8	Volume change due to hIEC at normative parameter values	ΔV_IEC_	571.23 ± 55.91	[cm^3^ h^-1^]	Fig 9, panels D-F

## Results

### Occurrence of Cool Nest Regions (CNRs)

At the nesting sites giant honeybees have to cope with the exposure to predators, but also with rain, wind and sun irradiation, which latter may lead to even excessively high temperatures. One of the most peculiar examples were nests under the hot tin roof of a college building in Assam, which heated up in the subtropical sun above 50°C (S1 Fig, panels A-B), with the risk of wax melting [60] and loosing the nest fixing., which affects the thermal properties of the cell building material. In contrast to Meliponines or Bombines, honeybees are unique among the social insects in using essentially unmodified wax for nest construction [60]. It is not known whether *A*. *dorsata*, nevertheless, mixes additives at extremely heat-exposed spots to increase the onset of wax melting.

Anyway, the colonies managed to cope with this critical situation by keeping the thermal effusivity [60–61] at the attachment of the comb extremely low; at least, as documented, the temperature at the nest surface was kept below 30°C, and therefore, they also succeeded to maintain the temperature milieu in the nest interior within acceptable limits [25–26, 55]. However, these conditions implicated the occurrence of a series of cool nest regions (CNRs) all over the bee curtain, which emerged and faded off in terms of minutes. To the contrary, even at the same daytime, honeybee nests in the cooler canopy of a 40 m high tree hardly showed any CNRs (S1 Fig, panel E).

For this study, CNRs have been detected on various nests (n_CNR_ = 207, n_nests_ > 20, n_days_ = 11) under different environmental conditions (Fig 2; S1 Movie). Although they occurred more often under higher ambient temperature (T_amb_), particularly in the afternoon, they were also observed under much lower T_amb_ in the early morning hours and during night-time (Figs 2–4). In the main experiment, their average numbers remained constant from the late morning up to 14.00 h, but then they markedly increased for two hours (Fig 4, panel B), proportional to ambient temperature (Fig 4, panel C).

### Temperature profile of an individual CNR

In Fig 5 the temperature profile of a sample CNR, was monitored regarding six pixel partitions between T_mid_ and T_min_ (Fig 5, panel B; n_ff_ > 7 000; for definition, see Methods and the scale in panels C-D). At the start of observation the temperature range in the entire A_env_ was T_max_—T_min_ = 1.988 ± 0.004°C (mean ± SEM; partial ranges: 0.3246 ± 0.0007°C; T_min_ = 31.97°C; T_max_ = 36.11°C), whereas the temperatures slightly decreased to the end of observation (T_min_ = 30.51°C; T_max_ = 34.54°C). The cooler partition, positioned near T_min_ (dark blue coding in Fig 5, panel E) made up less than 20% of A_env_, while the warmer partition positioned near T_mid_ (red coding in Fig 5, panel E) was about 50% of A_env_.

The monitoring of this CNR started at 15:32h, only minutes after its emergence, and continued over 30 min, when T_amb_ rose slightly from 29.2° to 29.5°C over 6 min and decreased by 1°C in the further 20 min (Fig 5, panel G). The performance of the CNR correlated with the ambience twofold: (a) the partial layers of the temperature profile, represented by pixel-related temperature values T_CNR_, graded with T_amb_ (Fig 5, panel H; e.g. partition 1: R^2^ = 0.7586, and partition 6: R^2^ = 0.7172) and (b), the T_CNR_ values were modified by the plateau of T_amb_ to form a second peak (Fig 5, panels F-G). In addition, the sample CNR shifted in upward-left direction (Fig 5, panels B-D, horizontal: Δ_r_POS_hor_ = -1.5 cm; vertical: Δ_r_POS_vert_ = +0.5 cm), along with a high scatter factor in the initial phase (Fig 5, panels C-D).

### Fanners at CNRs

**Fig 6:**
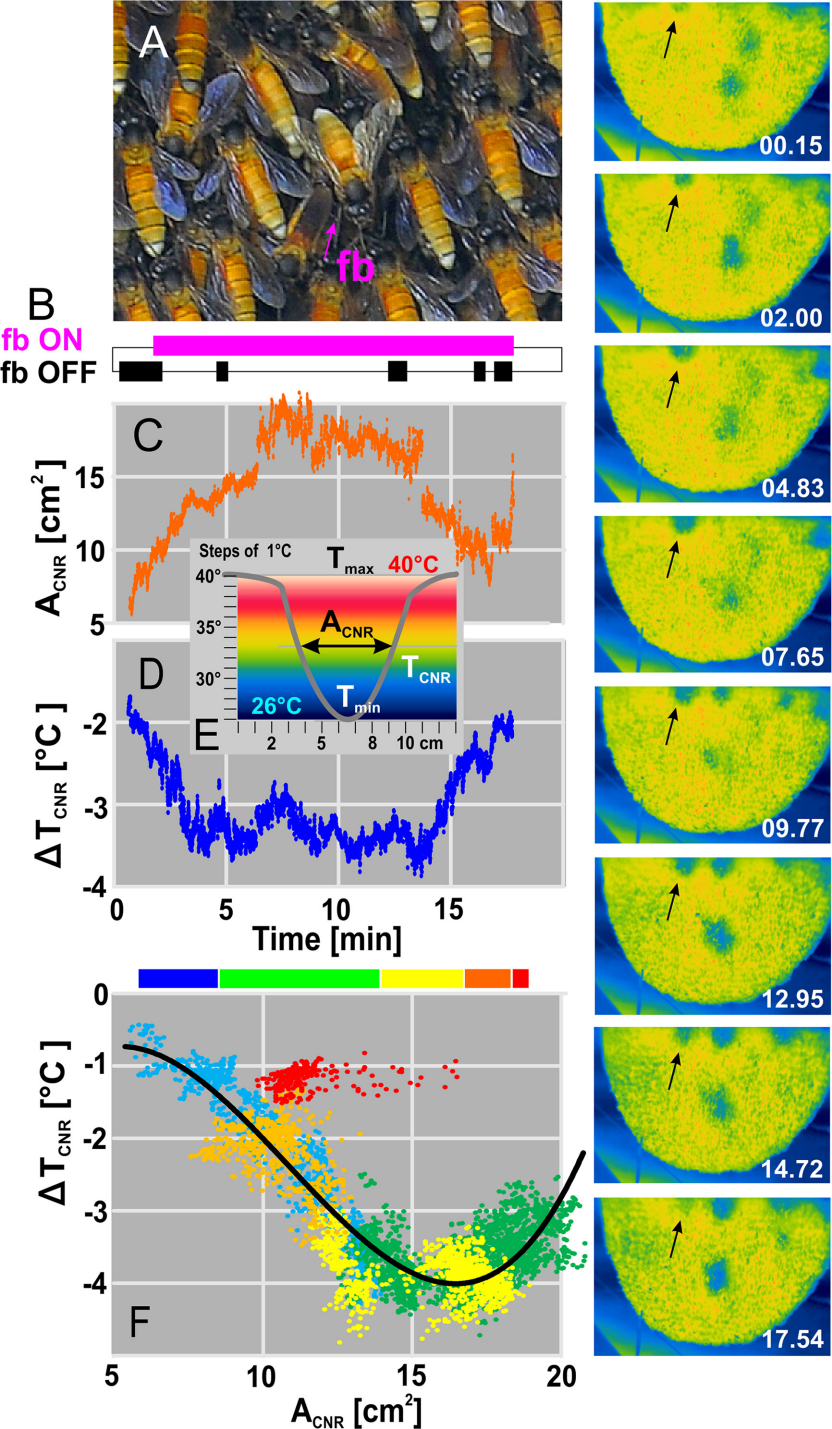
Size-temperature plots of a sample CNR. The black arrows in the IR images (“nest 02”) on the right side show the CNR selected; white text gives the relative time in min in decimal format from emerging (at 15:30 h) until fading off. (A) Close-up view with a fanning bee (fb, marked by lila arrow) positioned in the centre of the selected CNR. (B) Activity plot of fanning: lila: active fanning; black: “non-fanning” or “fanning bee is not visible”. (C) Time course of the CNR size (A_CNR_) in cm^2^. (D) Time course of differential CNR temperature (ΔT̃_CNR_, see Eq 4). (E) Sketch for the definition of the CNR parameters (T_max_, T̃_sink_, T_min_, A_CNR_ ≈ A_mid_) displaying 16 temperature partitions (ordinate) between 26° and 40°C. (F) Correlation between ΔT̃_CNR_ and A_CNR_ regarding six intervals of relative time: (a) blue, from 1.00 to 3.53 min (decimal format) after the start of observation; (b) green, 3.53–10.58 min; (c) yellow, 10.58–14.10 min; (d) orange, 14.10–16.22 min; (e) red, 16.22–16.91 min. The nonlinear display of the data (see black regression line for the time intervals blue to orange with R^2^ = 0.735) let assume that funnel control varies in several states: initially A_CNR_ grows larger while ΔT̃_CNR_ turns cooler (blue coded state), followed by a series of permutations (green and yellow coded state); before the CNR disappeared (red coded state) A_CNR_ varied around a constant magnitude of ΔT̃_CNR_.

In most cases when CNRs were monitored jointly in IR and HD from the first notice until their disappearance (n_CNR_ < 40; n_nests_ = 9) fanning bees were identified as positioned close to the CNRs (n_fb_ > 100). For example, in Fig 6, panels A-B and in S2 Movie the fanning bee was identified at the left rim of the CNR, while she characteristically kept the head away from the nest and the abdomen towards the nest surface, producing an air stream directed obviously towards the centre of this selected CNR.

The functional properties of CNRs were described frame wise by mid-based parameters (see Eqs 1–4) of temperature measures (T_mid_, T̃_sink_, ΔT̃_CNR_), gravity positions (X_sink_, Y_sink_) and size (A_mid_, A_CNR_). For the selected CNR (see schematics in Fig 6, panel E) the temperature range was 14°C (T_min_ = 26°C; T_max_ = 40°C), whereby its size (A_CNR_; Fig 6, panel C) stepped up in the first 7 min to its maximum of 20 cm^2^ to decrease slowly afterwards. At the same time, the differential temperature (ΔT̃_CNR_; Fig 6, panel D) decreased by 3.5°C within 3 min, remained fairly stable for further 10 min, and drifted back by 2°C to the surface temperature rapidly within 3 min. In this sample a fanner bee was active at the CNR, roughly over the entire filming session (Fig 6, panel B; S2 Movie). The regression between ΔT̃_CNR_ and A_CNR_ values shows that the initial main temperature drop (colour-coded as blue phase in Fig 6, panel F) correlated with the opening of the CNR, and that the final rise (colour-coded as orange phase in Fig 6, panel F) happened together with its closing. (However, there was an additional, very short re-opening in the later red-marked phase, see also Fig 6, panel C). Both the opening and closing phases are positioned at the same, left-sided branch of the correlation (Fig 6, panel F) while the central opening phase (at 4–14 min in Fig 6, panels C-D) led to a second positively sloped branch on the right side of the correlation.

In this example, the fanner bee was active throughout the presence of the CNR, explicitly also during opening and closing, but did not affect temperature nor size of the CNR. Therefore, it can be supposed that the dynamics of CNRs are controlled primarily by a collective of curtain bees located around CNRs rather than by fanners.

### Identification of the sources making up the temperature patterns in the nest surface of *A*. *dorsata*

A single bee is capable of heating up the thoracic muscles to temperatures much above T_amb_ [25–26, 48–56]. This is illustrated in *A*. *dorsata* from dancers and their followers during waggle dance (S1 Fig, panel C), or from water foragers prior to taking off (S1 Fig, panel D). The close-up image of the dancer’s episode (S1 Fig, panel C) also shows quiescent bees of the surface layer with uniquely cool body temperatures, whereas the cavities between these surface bees point to the warmer nest interior. This temperature gradient within the bee curtain can also be demonstrated when the nest surface was depleted exposing the sub-surface layer of quiescent bees below (e.g. by treatment with lavender oil: S1 Fig, panels F-G).

**Fig 7:**
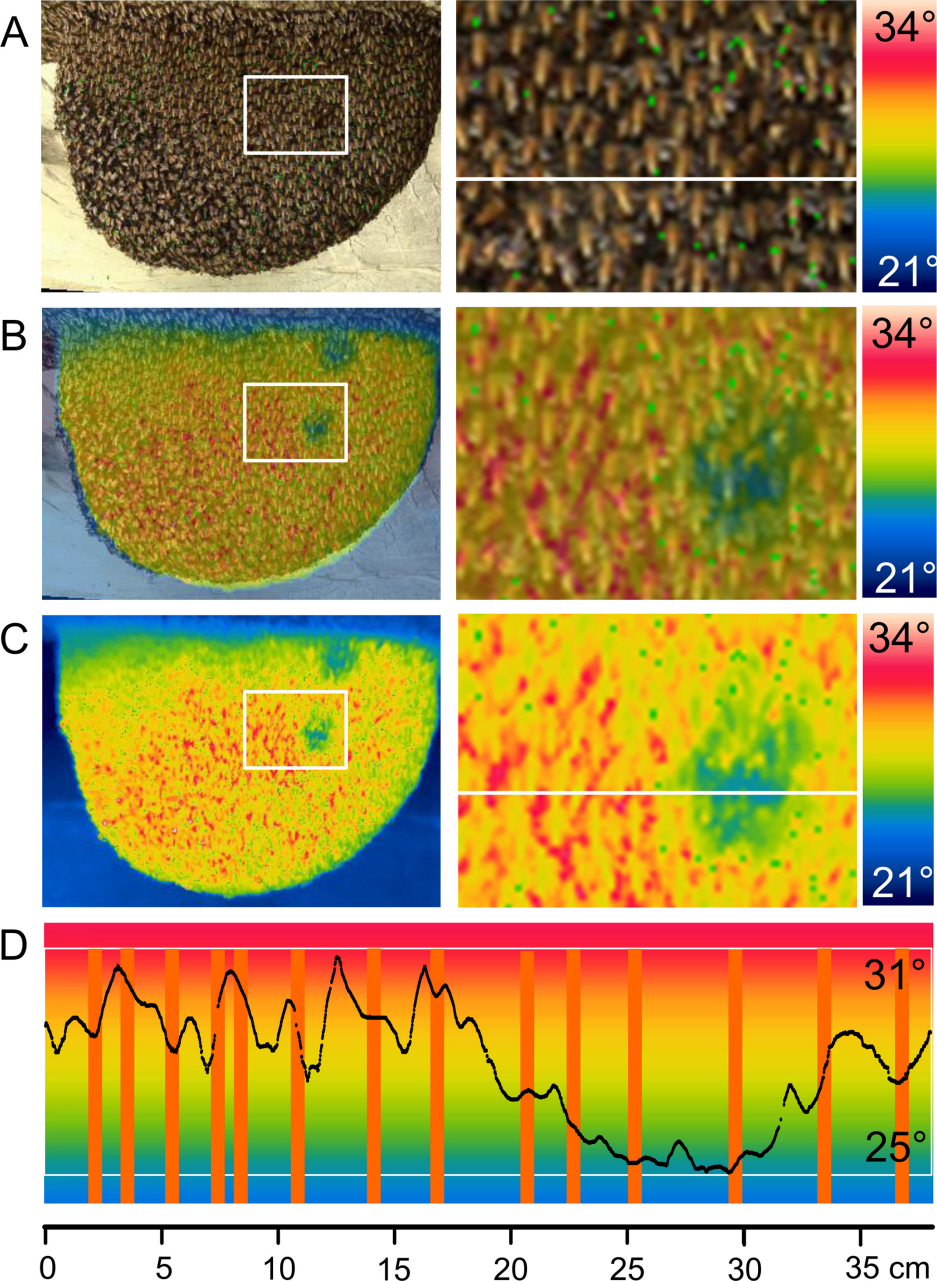
Identification of warm and cool zones at the surface of a giant honeybee nest. (A-C) IR overlays upon synchronously recorded HD images at different HD/IR transparency levels: panel A, 100/0; B, 50/50; C, 0/10 in per cent. Left side, overview of *nest 02*, white open inset rectangles regard to the close-up images on the right side, in which the small green spots mark the isotherm level (27.6° - 27.7°C); rainbow-900 scales (21° - 34°C) on the right side of the image panels. White horizontal lines in the right image panels (A, C) are detection lines with the length of 38 cm. (D) Black curve, temperature values [°C] scanned along the detection line in the IR image (panel C); the vertical orange bars give the positions of the bee bodies along this line as assessed by image analysis (panel A).

The spatial pattern of surface temperature was assessed along transect lines through CNRs and their warmer surroundings (Fig 7) in synchronously recorded HD and IR images, which allows to identify abdomens of the bees of the surface layer as cooler than the interstices between them. This example demonstrates how surface bees loop up the lower temperature from the ambience with the consequence that even in the centre of a CNR the bee bodies were slightly cooler at the surface than in the subsurface layer (Fig 7, panel D).

Consequently, there are two surmises to be made: (1) Together with the finding that T_CNR_ correlates with T_amb_ (Fig 5, panel H) evidence is strong that CNRs function as funnels through which ambient air flows into the nest, representing low-resistance gates for bundling in-going air streams.

(2) Without any airflow and at a given thickness of the multi-layered bee curtain, the interstices around a CNR are supposed to be affected more by the cooler ambience than by the warmer nest interior. Nevertheless, in all IR images these interstices were much warmer than expected (e.g. Fig 7; S1 Movie). Therefore, it is plausible to assume that these interstitial temperatures are caused by airflows by which warm, nest-borne air comes out, widely dispersed, through the mesh of the bee curtain around CNRs. Both surmises allow postulating the existence of a driven convection supporting respiration of the nest, by generating a cool airstream flowing inwards through CNRs and an outward nest-warm airflow through the mesh of curtain bees in the periphery of CNRs.

### Tracing a respiratory mechanism by the assessment of size and temperature of CNRs

**Fig 8:**
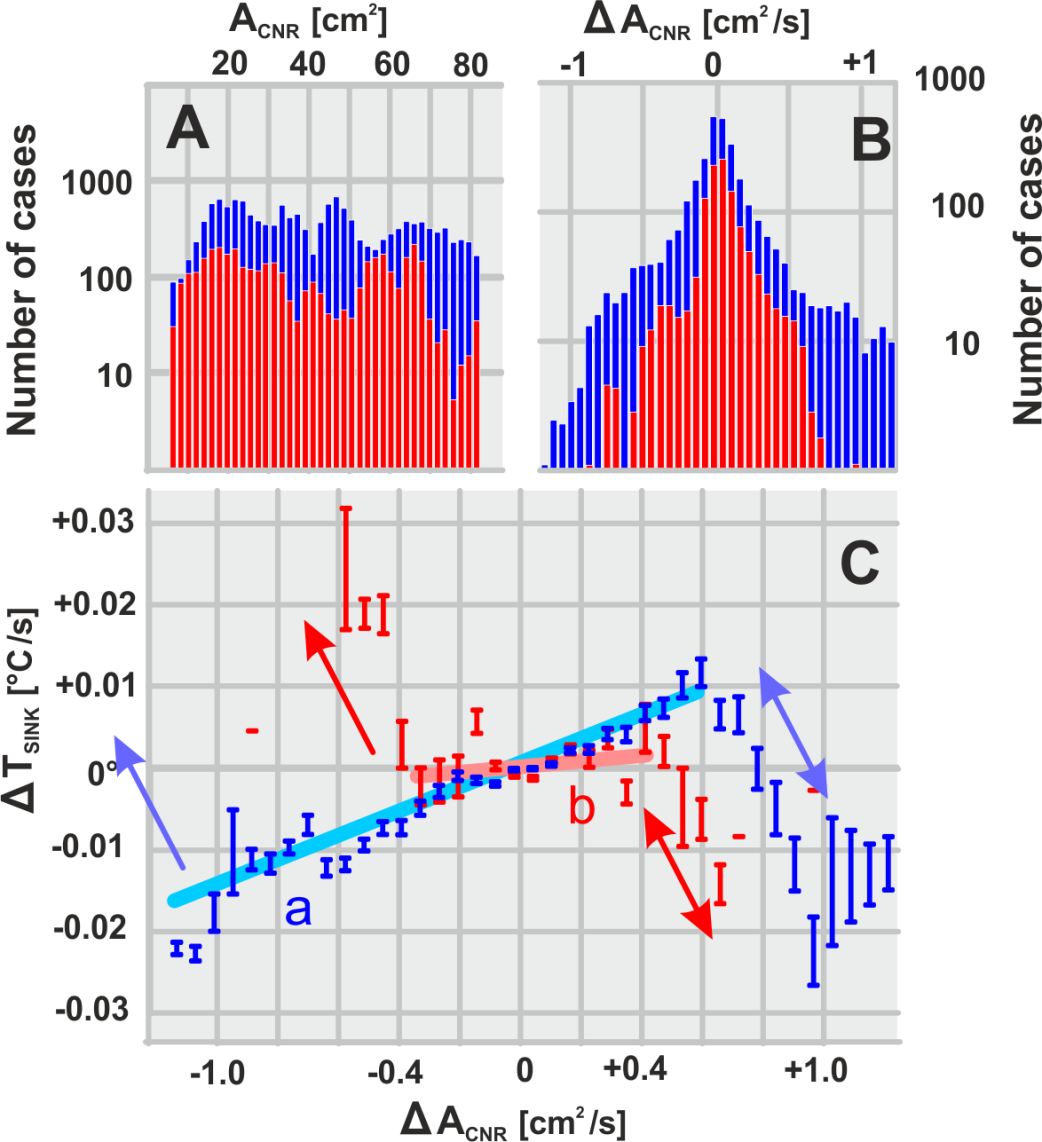
Differential size (ΔA_CNR_ /Δt)—temperature (ΔT̃_sink_ /Δt) values of six selected CNRs (“nest 02”). Correlation of the summarized values ΔT̃_sink_ and ΔA_CNR_ under checked fanning conditions; for the number of cases (= pairs of images), see panel B): blue colour codes refer to the “presence of actively fanning bees”; red codes refer to the state “without fanning”. Differential values (ΔT̃_mid_ /Δt, ΔA_mid_ /Δt) were assessed frame by frame over time intervals of 100 frames corresponding to Δt **=** 21.16 s. (A-B) A_CNR_ data were uniformly distributed, and ΔA_CNR_ data were gauss distributed. (C) Regression functions (a: blue-coded, “fanning” state with R^2^ = 0.9353; b: red coded, “non-fanning” state with R^2^ = 0.0617) concern the central region of both correlations. One-sided arrows on the left side of correlation symbolize “closing” reactions (data for fanning state are here outside the scope); double-sided arrows on the right side give “inhalation” activities (see Discussion for ventilation hypothesis). Vertical blue and red bars in panel C give the range of data within ± standard error.

The entire data set of the selected experimental nests (n = 9) comprised 334 synchronized sequences of IR and HD images. For the in-depth analysis described in this chapter only the dataset “09/nest 02”was used; it comprised a session over 13 days, from 9 am to 6 pm recording time each and delivered the highest abundance of CNRs compared to all other experiments. The differential data (ΔA_CNR_/Δt, ΔT̃_sink_/Δt) are plotted in Fig 8 and referred to time intervals of Δt = 21.16 s (corresponding to a sequence of 100 IR frames). Under both conditions, with and without fanning bees, the A_CNR_ data (Fig 8, panel A) were uniformly distributed (between 20 and 80 cm^2^), but their differential data (ΔA_CNR_/Δt, Fig 8, panel B) were gauss distributed (between +1.0 and -1.0 cm^2^/s). Under the presence of fanning bees the selected data sets (Fig 8, panels A-B) were five times larger than under non-fanning conditions. The majority of data, concerning e.g. 97.20% for fanning conditions (n_ff_ = 24 359 inter-frame intervals; Fig 8, panel C), referred to the central zone of the ΔA_CNR_ plots (detailed below as “scenario 1”) while the complimentary minority was positioned at the periphery of both, positive and negative abscissa branches of ΔA_CNR_ data (“scenario 2”).

#### Scenario 1: Positive correlation between changes in CNR size and temperature

Under fanning condition (Fig 8, panel C, blue symbols) a smooth positive gradient was displayed centrally [-1.20 > ΔA_CNR_/Δt > +0.60 cm^2^/s]. It means that increasing CNR temperature (+ΔT̃_sink_) correlates with increasing CNR size (+ΔA_CNR_), which is, however, the same to say that a decrease in CNR aperture is associated with a cooling effect; e.g., for ΔA_CNR_ /Δt = -1.0 cm^2^/s the temperature decreased by ΔT̃_sink_ /Δt = -0.0111°C/s. In physics, this effect is known as Venturi effect; it is the consequence of the Bernoulli equation [61–62] that the temperature in an ideal gas decreases with increasing flow speed. With other words: if there exists a current flow through an aperture, any restriction of this aperture would increase the speed of the flowing medium and decrease its temperature.

In the case of CNRs, however, this Venturi effect is practically ineffective for any cooling effect of the nest interior, essentially because of the small magnitudes, but it still evidences the existence of a dynamically controlled air stream and therefore, once again, the funnel property of CNRs (illustrated by the blue regression line [a] in Fig 8, panel C: R^2^ = 0.9353, concerning 97% of data). Interestingly, the Venturi effect cannot be traced, at least not in the summarized data, for non-fanning conditions (red regression line [b] in Fig 8, panel C: R^2^ = 0.0617). This could mean that CNRs have funnel properties mainly under the assistance of fanning bees. (However, in the separate analysis of a series of CNRs, e.g. in S2 Fig, subpanels d, at least two out of six samples do show the Venturi effect even without the presence of fanning bees).

#### Scenario 2: Negative correlation between changes in CNR size and temperature

Theoretically, a negative correlation between differential data of size (ΔA_CNR_) and temperature (ΔT̃_sink_) can be explained by at least four different aspects: by an adiabatic process, by evaporation, by closing funnels, and by opening funnels.

(a) In adiabatic processes, a medium flowing through a physical tube is cooled by opening it and warmed up by constricting it. In nature this typically happens under foehn conditions by which air pressed over mountain ridges [63–64] is cooled down to its adiabatic dew point when rising through orographic lifting up, and is warmed up by adiabatic pressure on the leeward side of the ridge. Adiabatic processes are also utilized technically, e.g. in snow guns where water and pressurized air are forced through a narrow tube to be emitted into the open, which transforms liquid water into snow [65]. CNR funnels would, however, hardly comply with adiabatic cooling processes, as they would require extremely high energy levels concerning airflows and abruptly opening gates.

(b) In evaporation, cooling effort grades negatively with the size of a humid area. To support the *evaporation hypothesis* for CNRs, water must be delivered to drive evaporation. Water could be brought to the CNRs, e.g. by water foragers, to suspend droplets of water. Rapid evaporation could be established by gobbetting behaviour of such water carriers, by which they repeatedly extend and contract their probosces, eventually pressing drops of water from the mouths into a thin film [25], and re-ingest them after evaporative cooling. Theoretically, curtain bees may also contribute in such cooling at CNRs, if they collectively regurgitate watery honey [25], but they could also speed up this evaporation as fanners by producing air streams towards the CNRs. Fact is, however, no water carriers had been observed at CNRs up-to-date, and even more important, the size changes (ΔA_CNR_) correlate negatively with temperature changes (ΔT̃_sink_) independently from the visible presence of fanning bees (at least for ΔA_CNR_ > +0.40 cm^2^/s; Fig 8, panel C).

(c) The negative correlation in the changes of CNR size and temperature on the left-side branch of Fig 8, panel C (with ΔA_CNR_ /Δt < 0) refers to abrupt increases of T̃_sink_, possibly up to T̃_surr_, while the size A_CNR_ may have diminished to zero (Fig 8, panel C). This constellation is nothing else than the closing of CNR funnels, which, astonishingly, occurred under non-fanning conditions at smaller |ΔA_CNR_|/Δt values than under fanning conditions (which were in Fig 8, panel C even outside the displayed data range). Thus, fanning behaviour supports the funnel attributes of CNRs by enlarging the range of operation.

(d) The relatively large but rare (Fig 8, panels A-B: < 3% of the data) opening events of CNRs (ΔA_CNR_ /s > +0.60 cm^2^/s; Fig 8, panel C) also graded negatively with the ΔT̃_sink_ /s values. Their repetitive occurrence can be plausibly explained with the assumption that CNRs operate as convection funnels in nest ventilation. Combined with an “inhalation-exhalation cycling” (IEC) activity of the bee curtain, the opening of CNRs leads to a rapid “inhalation” of cooler ambient air and thus to a negative correlation between ΔT̃_sink_ and ΔA_CNR_.

### Tracing potential “respiration” movements in the bee curtain

The performance of such hypothetical CNR-based ventilation in an *A*. *dorsata* nest is determined by the assumed funnel properties of CNRs and by a proposed IEC process. In the “inhalation” phase, the lumen between comb and inner surface of the bee curtain must be widened out, which can be only generated by muscle force. As potential candidates, curtain bees in the nest interior positioned around CNRs may synchronously stretch the extremities pushing the bodies away from the comb. Such local increments of the interior lumen would cause minute, but assessable dislocations of the comb (ΔZ_comb_ << 1 mm) and of the bee curtain on both sides of the comb from the direction of gravity. Hereby, the comb and the contralateral part of the bee curtain would be shifted away from the stationary LDV, while the ipsilateral part of the bee curtain would be driven towards the LDV (see the schematics in S3 Fig, panel B). In the “exhalation” phase, the relaxation of the “stretcher” bees could reposition the comb and both parts of the bee curtain to the direction of gravity, to the original quiescent, “non-ventilatory” state (S3 Fig, panel A), narrowing again the inner lumen of the affected, ipsilateral nest side.

**Fig 9:**
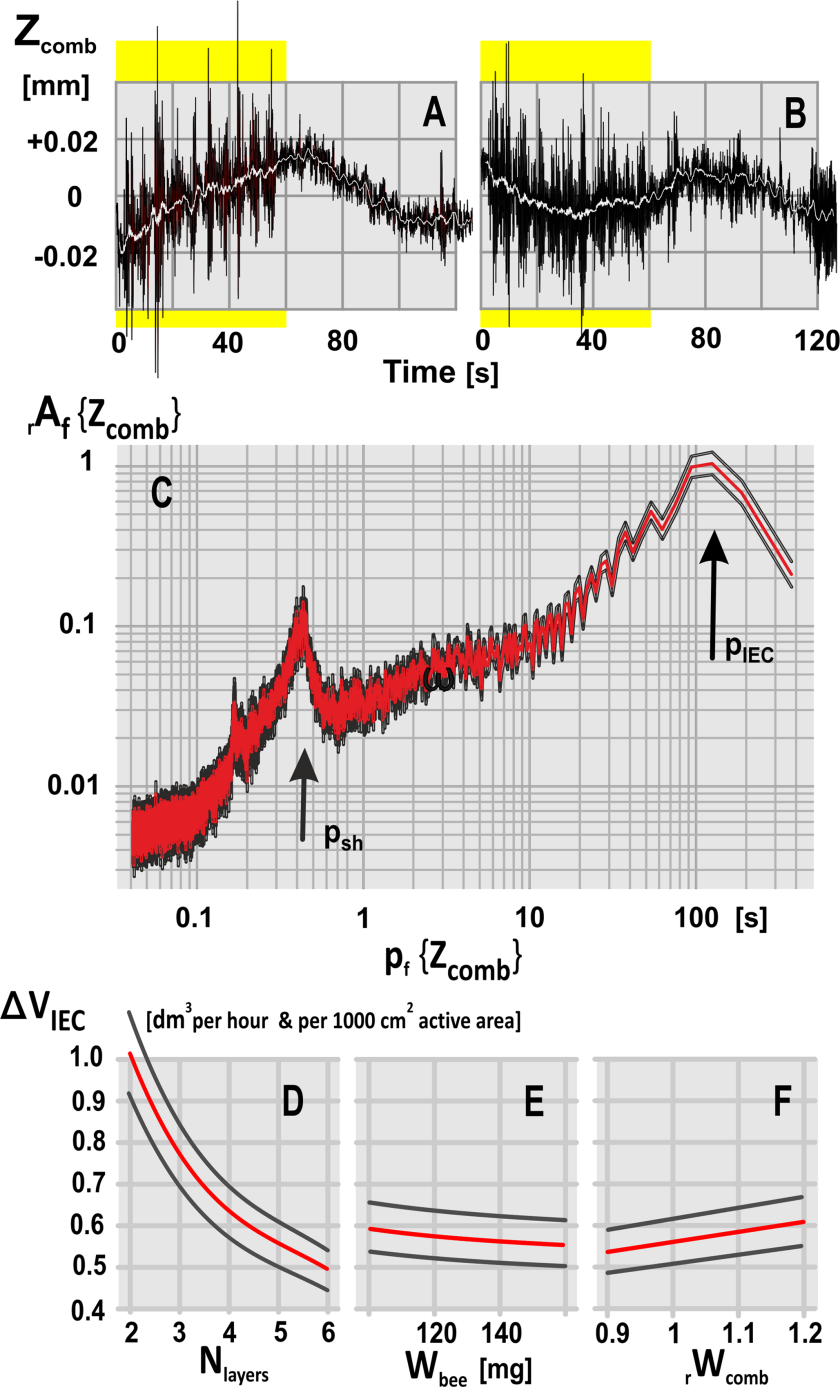
Comb dislocations in a giant honeybee nest during shimmering and quiescence. (A-B) Two LDV records of comb dislocations (Z_comb_; cf [30], scaled in mm/s; see Methods and S3 Fig). Black curves, unfiltered data; white superposed curves give low-frequency components defined as moving average values (±50 frames with interframe intervals of Δt_ff_ = 20 ms); yellow background marks the first 60 s of observation, in which shimmering waves had been provoked by dummy wasp presentation [30]. (C) FFT-diagram: abscissa, the periods of partial frequency components of comb dislocations p {Z_comb_} in s; ordinate, the relative amplitude of these frequency components rA_f_ {Z_comb_}. The period of the main low-frequency component presumably caused by “inhalation / exhalation cycling” (IEC) was assessed at p_f_ {Z_comb_} = p_IEC_ = 109.96 s (marked by the black arrow in panel C), as compared to p_sh_, the period of oscillation provoked by shimmering (cf. [30]); (D-F) Mathematical model estimating the hourly change in volume of gas exchange affected by IEC according to the ventilation hypothesis (ordinate: ΔV_IEC_ [dm^3^ h^-1^]). In this model, the size of the nest area affected by ventilation was chosen as 1 000 cm^2^ corresponding to an area with a radius of 17.85 cm around the respective CNR funnel. The volume of gas exchange is dependent on three mass parameters of the bee curtain (number of bee layers N_layers_: panel D; individual weight of a honeybee W_bee_ in mg: panel E) and of the comb (the relative weight of the comb area rW_comb_: panel F). Red curves, mean values; black curves, range of mean errors; n_LDV_ = 23 LDV episodes. Normative base data (see Tables 1–3): density of bees in the bee curtain = 1 bee per cm^3^; density of cells at both comb sides = 787 / dm^2^ [72]; mass of the wax = 24.11 g dm^-2^ [72]; this adds up to a mass of W_comb_ = 134.29 g /dm^2^ comb area with larvae-containing cells (corresponding with rW_comb_ = 1.0 in panel F).

The efficiency of such hypothetical ventilation depends on the rhythmicity of the respiratory movements (at the frequency ζ_IEC_) and by the magnitude of the air filling per cycle (ΔV_IEC_/Δt). Both values can be estimated on the basis of LDV data (Fig 9) together with the definition of nest parameters (Tables 1–3; Eq 5), such as the properties of the comb (mass of the comb per dm^2^, the density of cells, the mass of a single larva) and the arrangement of the bee curtain (the mass of a single bee, density of bees, number of bee layers on each side of the nest).

#### Assessment of the rhythmicity of respiratory (IEC) movements

In the experiment, the LDV records reveal dislocations of the comb (ΔZ_comb_) in two phases (n_LDV_ = 23; Fig 9, panels A-B; [30]): (a) during the presentation of a dummy wasp [28–31] provoking shimmering waves (sh), and (b) under quiescent conditions, i.e. without the presentation of the dummy wasp. Comb dislocations as caused by the shimmering activity can be identified at periods of < 1s (Fig 9, panel F: FFT peak marked by the p_sh_ arrow). The marked dislocations at much lower frequencies as disclosed by the FFT peak at p_IEC_ = 109.96 ± 0.55 s (Fig 9, panel C; considering the frequency band of 0.0025–0.1 Hz) do not represent any systemic LDV drift (which has already been corrected in the pre-assessment phase [30]), nor were they caused by external air convection (the nest was protected by linen curtains in the hotel bay, see S1 Fig). The only possibly explanation for these low frequency comb movements is to assume cycling respiratory movements due to nest ventilation. This view is supported by the IR data (S2 Fig, subpanels b) that most of the CNRs exhibited rhythmic openings and closings, alike the LDV records, in periods of minutes.

#### Volume changes affected by the hypothetical ventilation

Any dislocation of the nest triggered by external or internal forces concerns the comb and both sides of the bee curtain. In detail, if the ipsilateral side of the bee curtain with the mass m_1_ = M_bc_ipsi_ is dislocated from the comb (as a physical pendulum against gravity), also the opponent, conjoined masses of the comb plus the non-affected (contralateral) part of the bee curtain (m_2_ = M_comb_ + M_bc_contra_; S3 Fig; Table 2, Pos 4) are dislocated from the line of gravity at the same time. The resulting dislocation of the affected part of the bee curtain (ΔZ_bc_ipsi_) can be calculated considering the dislocation of the comb ΔZ_comb_ as assessed by LDV and both mass components (m_1_, m_2_) by applying the principle of the conservation of momentum and kinetic energy in coupled physical pendulums [66] (Eq 5).

$$\Delta {\text{Z}}_{\text{bc}\_\text{ipsi}}=\Delta {\text{Z}}_{\text{comb}}\frac{{\text{m}}_{2}}{{\text{m}}_{1}}$$ (5)

The mean dislocation of the comb at the cycling period p_IEC_ was estimated on the basis of the LDV records at the maximum of average dislocation values (ΔZ_comb_ = 58.58 ± 5.73 μm; n_LDV_ = 23; cf. Table 3, Pos 1–4). Under normative nest conditions (defined in Tables 1–3) the mass of the bee curtain at one side was 70.71 g / dm^2^ and the mass of the comb was 134.29 / dm^2^ resulting in a mass relation of m_2_ / m_1_ = 205.00 g / 70.71 g = 2.8992 (Table 2; Eq 5; see S3 Fig for schematic representation). Therefore, the real dislocation at the affected comb side during the “inhalation” phase can be estimated by ΔZ_bc_ipsi_ = 169.84 μm (Table 3, Pos 4), which corresponds well with the hypothetical range, the bees activated at the inner surface of the bee curtain could stretch their extremities.

On the basis of this dislocation value (ΔZ_bc_ipsi_), applied to an affected area of 1 000 cm^2^ around a CNR (r = 17.85 cm), the support of fresh air from the ambience would amount to an hourly volume of 571.23 ± 55.91 cm^3^ (n_LDV_ = 23; Table 3, Pos 8). However, CNRs can here only run as potential “breathe-in” gates if the airway resistance inside the funnel (R_CNR_) is much lower than that of the neighbouring mesh of the bee curtain (R_mesh_). In detail, the IR images document just this: CNRs behave in such a hypothetical “inhalation” period like nostrils in mammals (with R_CNR_ << R_mesh_). In the consecutive”exhalation” phase, the relaxation of the affected curtain bees brings then, simply by gravity, all dislocated masses (m_1,_ m_2_) back into the vertical direction, pressing nest-borne air outwards through the diffuse mesh of the bee curtain (illustrated in Fig 7, panels C-D and in S1 Movie). Therefore, CNRs may operate here likewise as one-way valves giving access for inbound airflow rather than representing a homogenous, leaking part of the bee curtain.

## Discussion

### Occurrence of CNRs

Giant honeybee nest regularly display on their surface small (10–100 cm^2^) regions, which are cooler (<-2°C) than their adjacency (S1 Movie). Such cool nest regions (CNRs) occur day and night, more often at higher ambient temperatures (typically at T_amb_ > 27°C: Figs 3–4), at central regions of the nest [6–13] but also at those zones where the nests are attached to the substrate (Figs 2 and 3; S1 Fig). They are found in fully established nests containing brood as well as in migration bivouacs (Fig 2; [8]). Their occurrence in such temporary nest clusters implies their importance for maintaining the milieu inside the nest, not solely driven by the goal of brood incubation (*Apis dorsata*: [14–15]; general: [16–26]).

### Fanning at CNRs

At most CNRs, solitary fanning bees were detected (n_CNR_ = 60) in a characteristic body alignment, by pointing their heads away from the nest and producing air streams, which were directed towards the centre of CNRs (Fig 6, panel A; S2 Movie). Fanning in *A*. *dorsata* can generally be observed in various contexts: it assists in evaporation of water droplets delivered by water foragers and pushes away raindrops from the nest surface [6], but it also occurs, though in a confined way, during shimmering [7–8, 27–33].

In European honeybees (*A*. *mellifera*) fanning is found mainly at the entrance hole of the hive, in “Sterzeln” [21–25, 46–47] and ventilating [40–58]. In both behavioural contexts, the bees hold themselves tight with their extremities on the underground, point with the heads towards the nest outlet, and produce airstreams directed away from the hive, which drives air out of the hive. During “Sterzeln”, the bees release Nasonov scent [67–68] by splaying their abdomens in a stationary posture with elevated abdomen and exposed Nasonov scent glands. Nasonov pheromone particularly provides information for the homing foragers to find the mother nest [8, 68]. In *A*. *dorsata*, the release of clouds of Nasonov pheromone around the nest during shimmering [27–33] encourages other nest mates to participate in stationary shimmering position preventing their transmutation into flying guards.

In *A*. *mellifera* nests, ventilation is conjoined with pumping out nest-borne air through the entrance hole [40–56]. Fanners of the Indian honeybee *A*. *cerana*, however, align their bodies with the heads away from the nest [57–58], which presses air into the nest entrance. Most likely this is the evolutionarily older way to drive ventilation in honeybees, which is utilized even by cavity nesting species (provided the nest borne air can flow out under this pressure from outside through leaking hive structures). This could explain why also the fanners of the also evolutionary older, open-nesting giant honeybees direct their air streams towards the bee curtain (Fig 7, panel A; S2 Movie). As we have observed, this fanning in *A*. *dorsata* was definitely not escorted by Nasonov scenting and was also not linked to any evaporative process, neither mediated by water foragers nor by curtain bees as potential honey storers. Moreover, it is physically impossible that airflows initiated by such fanners towards the nest surface could effectively ventilate the interior of *A*. *dorsata* nests through CNRs.

### Funnel and ventilation hypotheses

The occurrence of CNRs correlates with ambient conditions (Fig 4), whereby CNRs, nevertheless, possess autonomy in their spatial dynamics (Figs 5 and 6). The data bring reliable evidence that CNRs do not effectuate evaporative cooling but do have funnel functions (Fig 5, panel H; Fig 8, panel C). The performance of CNR funnels is obviously supported by fanning bees (Fig 6, panel A), they are likely to enlarge the operational “Venturi” range [61–62] by stimulating the initiation and maintenance of CNRs (Fig 8, panel C) when pushing air from the cooler ambience towards CNRs.

Here, the formation of CNR funnels prompts the questions how the air streams through CNRs are directed inwards, how they are generated and how they ventilate the nest. The findings of this paper (Figs 6–8) definitely exclude fanning activity as the main motor for ventilation without any primary role in opening and closing of CNRs (Fig 6; Fig 8, panel C). They support the view, however, that *A*. *dorsata* colonies have obviously found a more forceful solution to safeguard aeration of the nest interior than pushing ambient air into the nest by fanning. The most plausible explanation is in support of the ventilation hypothesis, which presumes the existence of “inhalation-exhalation” cycling (IEC). Ventilation may happen here quite analogous to the abdominal respiration in mammals [69], where the muscular force of the diaphragm rhythmically enlarges the lung volume, which leads to a pressure fall in the lungs and instils an airflow inwards through the nostrils.

In *A*. *dorsata* nests an analogous process could suck fresh air from the ambiance into the nest, promoted by CNR funnels as low-resistance gates for air streams. The pumping mechanism behind this “breathe-in” probably is due to transient and synchronously forced actions of a collective of curtain bees around CNRs. According to this surmise, such bees could stretch their extremities against the comb to arch the bee curtain slightly outwards, which enlarges the nest lumen at the comb site, causing a fall of pressure herein. In the consecutive “exhalation” phase, the same bees, previously active in pushing themselves from the comb by muscle force, would relax. This would drive the bee curtain back to the comb by gravity, pressing the warm, residual, nest-borne and CO_2_-enriched air outwards through the diffuse leaking mesh structure of the bee curtain. Such transient ventilation should occur rhythmically, and should be not necessarily coupled with the presence of active fanners.

These surmises of the ventilation hypothesis are verified by a series of quantitative observations: (a) Funnel functions are substantiated for CNRs by the Venturi principle [61–62] (Fig 8, panel C; S2 Fig). (b) The inwards direction of the airflows through CNRs is inferred from the body alignment of the fanners at CNRs (Fig 6, panel A) and by the strong influence of the temperature profile of the CNRs with ambient temperature (Fig 5, panel H). (c) IR imaging evidences CNR funnels as “inhalation” tubes (through which air flows inwards, analogously to the mammal nostrils in the “cool” phase) rather than as “exhalation” outlets (Figs 6 and 7). (d) Cooling by opening happened at CNRs in the presence of fanners but also without them (ΔA_CNR_ > +0.40 cm^2^/s; Fig 8; S2 Fig). (e) Fanning stimulates the nest for voluminous “breathe-ins”, which is documented by the expansion of the “cooling” range by dilating CNR funnels under fanning. (f) Rhythmicity of ventilation is documented by two different methods of data assessment, both revealing cycling in periods of 1–2 min: in IR imaging, by the mostly pulsating time courses of the CNR apertures (e.g. in the A_CNR_ data of S2 Fig, subpanels c), and in the LDV signals, by the low-frequency rhythm of comb dislocations at the period length of p_IEC_ (Fig 9, panel C). (g) The hypothetical “exhalation” process leads to a leaking out of warm air through the mesh of the bee curtain, which is factually traced in the sequences of IR images (Fig 7; S1 Movie). Considering that the interstices between bees at the nest surface are practically exposed to ambience, these cavities, which allow the view on the subsurface layer of the bee curtain, are much warmer than expected without any outward flow of nest borne air.

Lastly, the mathematical model (Fig 9, panels D-F) allows to estimate the capacity of such a hypothesized IEC process in nest ventilation: at normative conditions (see Tables 1–3 for definition), the hourly aeration of the nest through CNR funnels amounts at least to more than 0.5 dm^3^ fresh air per 1 000 cm^2^ nest area (Table 3, Pos 5), in which the lumen between bee curtain and comb is rhythmically expanded. An *A*. *dorsata* colony may raise this hourly volume of gas exchange simply by increasing the number of CNRs in the nest to meet the needs even under the higher ambient temperatures of a subtropical mid afternoon (e.g. Figs 2 and 3; S1 Movie).

### Conclusive remarks

The bee curtain of an *A*. *dorsata* nest possesses five to seven layers of bees and thus provides a relatively dense and insulating cover for the protection of the brood against environmental curtailing, but keeps off venting it without additional strain. The CNR funnels, so far as known up-to-date, are the only way to provide the brood under undisturbed conditions with both homoeothermy and ventilation with fresh air.

Hereby, the need of ventilation in honeybee nests is considerably high: even quiescent honeybees consume due to their high metabolism rate notable amounts of oxygen (e.g. *A*. *mellifera* at 35°C: 5.55 μl O_2_ min^-1^ per individual [70–71]), even linearly increasing at falling ambient temperature, and produce hereby a considerable amount of CO_2_ [71]. Honeybees are also highly susceptible to CO_2_ gradients [70]: this is shown in *A*. *mellifera* colonies, which intensify fanning inside and outside the nest with increasing partial pressure of CO_2_ in the nest [71]. And it is also observed in *A*. *dorsata* nests, in context with the occurrence of CNRs, which emerge not only under higher ambient temperatures in the early afternoon but also in morning and evening hours when the ambience is much cooler (e.g. during November in Chitwan with T_amb_ = 23°-24°C; Fig 2, panel C; Fig 3). Evidence is therefore strong that CNR*s* depict low-resistance convection funnels not only for maintaining inner nest homoeothermy but also for restoring fresh air in the nest.

## Supporting Information

S1 Fig IR displays of *Apis dorsata* nests and of individual bees. (A) Assemblage of nests attached to a tin roof of a college building (West Assam, 1998). (B) Four IR images of one and the same nest documented within one hour of observation: the vertical line structures regard to the ripples of the tin roof; a series of CNRs were developed which changed in number, size and position. (C) Close-up views from the surface of an experimental nest revealing a dancing forager bee and her followers with hot thoraces and cool abdomens, whereas neighbouring quiescent surface bees had uniquely cooler bodies at ambient temperature; the yellowish areas around some surface bees regard to interstices and allow the view at the warmer, deeper layers of the curtain. (D) A single water forager, some seconds before taking off from the water place with the characteristic heated-up thorax. (E) Nests in the canopy region of a big tree without any CNRs (Assam, 1998). (F-G) Experimental nest before (F) and during (G) manual treatment with lavender oil which depleted the surface layer; the bees of the surface layer were urged to walk aside. The higher temperature of this lower exposed layer comes from the nest interior (Chitwan, Nepal 2010). Insets give the temperature scales of the images.
(TIF)

S2 Fig CNR size and temperature profiles. Six CNRs (A-F) were selected for evaluation (IR images at the top row) concerning nest 02 (Chitwan, Nepal) within an observation window of 30 min; duration of the time intervals of the individual CNRs: t_OBS_ = 16.03 ± 2.80 min (mean ± SEM, n_CNR_ = 6). Each group of the diagram panels (A-F) display five sub-panels (a-e): (a) The distributions of A_CNR_ values (abscissa: A_CNR_ [cm^2^]; ordinate: number of cases corresponds with the number of IR inter-frame intervals; note the logarithmic scale). (b) The time courses of the size of CNR in the observation interval (abscissa: time [min]; ordinate: A_CNR_ [cm^2^]). (c) Status of fanning: blue colour [F]: the manual evidence of the existence of at least a single fanning bee close to the CNR; red coding [non-F]: no fanning bee was discriminated. (d) The distribution of ΔA_CNR_ values per CNR (abscissa: ΔA_CNR_ [cm^2^/s]; ordinate: number of cases). (e) Correlation of ΔT̃_sink_/s values [°C/s] (ordinate) in relation to ΔA_CNR_ /s values [cm^2^/s] (abscissa); differences were assessed over 100 frames corresponding to a discrimination period of 21.16 s.
(TIF)

S3 Fig Schematics to define dislocations of bee curtain (ΔZ_bc_iosi_) and comb (ΔZ_comb_) during the hypothetical IEC in a giant honeybee nest. A, quiescent, “non-ventilatory” phase; B, “inhalation” phase. Red areas symbolize the bee curtain, the grey areas the comb; thin black horizontal lines show that the bees of the inner surface of the bee curtain contact the comb with their extremities; longer lines refer to stretched extremities; horizontal orange bar is a wooden rod which was stuck through the comb and the ipsi- and contralateral parts of the bee curtain (bc_ipsi, bc_contra). The LDV ray was reflected on the ipsilateral plane end of the rod. Black vertical arrows give the direction of gravity (d_g_). The dislocation of the comb (ΔZ_comb_) from the direction of gravity was measured with LDV and the dislocation of the ipsilateral bee curtain **(**ΔZ_bc_ipsi_), locally around the convection funnel, was calculated according to Eq 5.
(TIF)

S1 Movie Occurrence of CNRs. IR monitoring of experimental “nest 02” (cf. Fig 1) through 33 min at 3.9427 Hz. The temperature scale on the right is fixed between 26° and 40°C, whereby the range of the actual temperature pixels is displayed by the small bar between the scale partition and the rainbow-900 palette. The black bar at the bottom displays the relative time of the image noted in minutes. The blue-green areas represent CNRs while the tiny red spots regard to the interstices between the bees of the surface layer; according to the *ventilation hypothesis* they document the “exhalation” phase when nest-warm air is pressed out through the diffuse mesh of the curtain (cf. Fig 7).
(MP4)

S2 Movie Fanning bee at a CNR. In the quiescent region of the bee curtain, peripheral to the mouth zone [6–8, 12], most of the bees are positioned in a vertical body alignment, with heads up and abdomens down. However, some bees display postures with the head pointing away from the vertical surface of the nest. These bees are potential fanners, most of them were identified near or at CNRs. This bee displayed in the mid of the image was fanning throughout nearly the entire observation session (cf. Fig 6, panel A).
(MP4)
