# Supplementary figures and images for: Evidence for Ventilation through Collective Respiratory Movements in Giant Honeybee (Apis dorsata) Nests
Source: PLoS One. 2016 Aug 3;11(8):e0157882. doi: 10.1371/journal.pone.0157882 (PMC4972441; doi:10.1371/journal.pone.0157882)

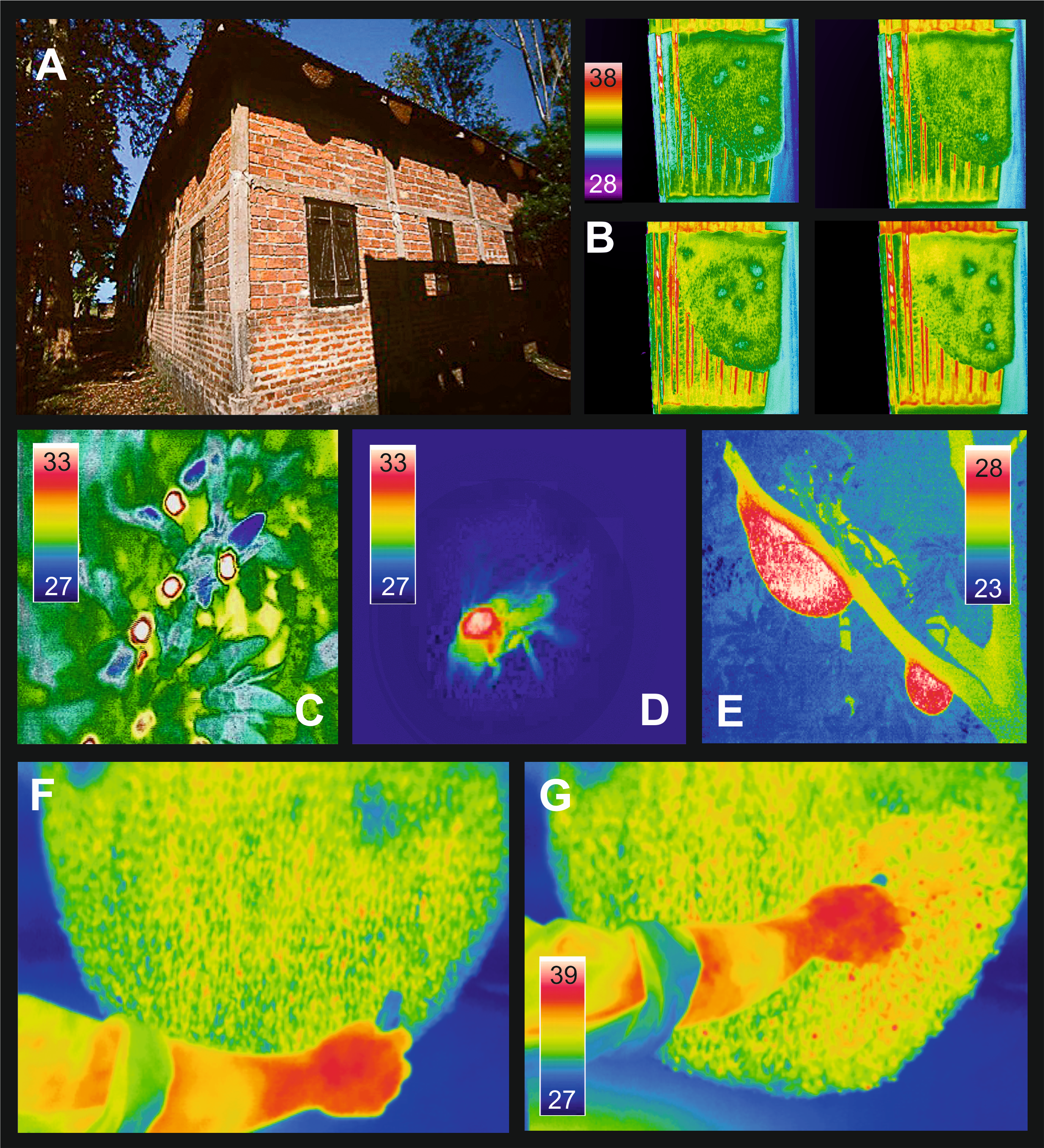

Supplement: S1 Fig — (A) Assemblage of nests attached to a tin roof of a college building (West Assam, 1998). (B) Four IR images of one and the same nest documented within one hour of observation: the vertical line structures regard to the ripples of the tin roof; a series of CNRs were developed which changed in number, size and position. (C) Close-up views from the surface of an experimental nest revealing a dancing forager bee and her followers with hot thoraces and cool abdomens, whereas neighbouring quiescent surface bees had uniquely cooler bodies at ambient temperature; the yellowish areas around some surface bees regard to interstices and allow the view at the warmer, deeper layers of the curtain. (D) A single water forager, some seconds before taking off from the water place with the characteristic heated-up thorax. (E) Nests in the canopy region of a big tree without any CNRs (Assam, 1998). (F-G) Experimental nest before (F) and during (G) manual treatment with lavender oil which depleted the surface layer; the bees of the surface layer were urged to walk aside. The higher temperature of this lower exposed layer comes from the nest interior (Chitwan, Nepal 2010). Insets give the temperature scales of the images. (TIF) [file pone.0157882.s001.tif]

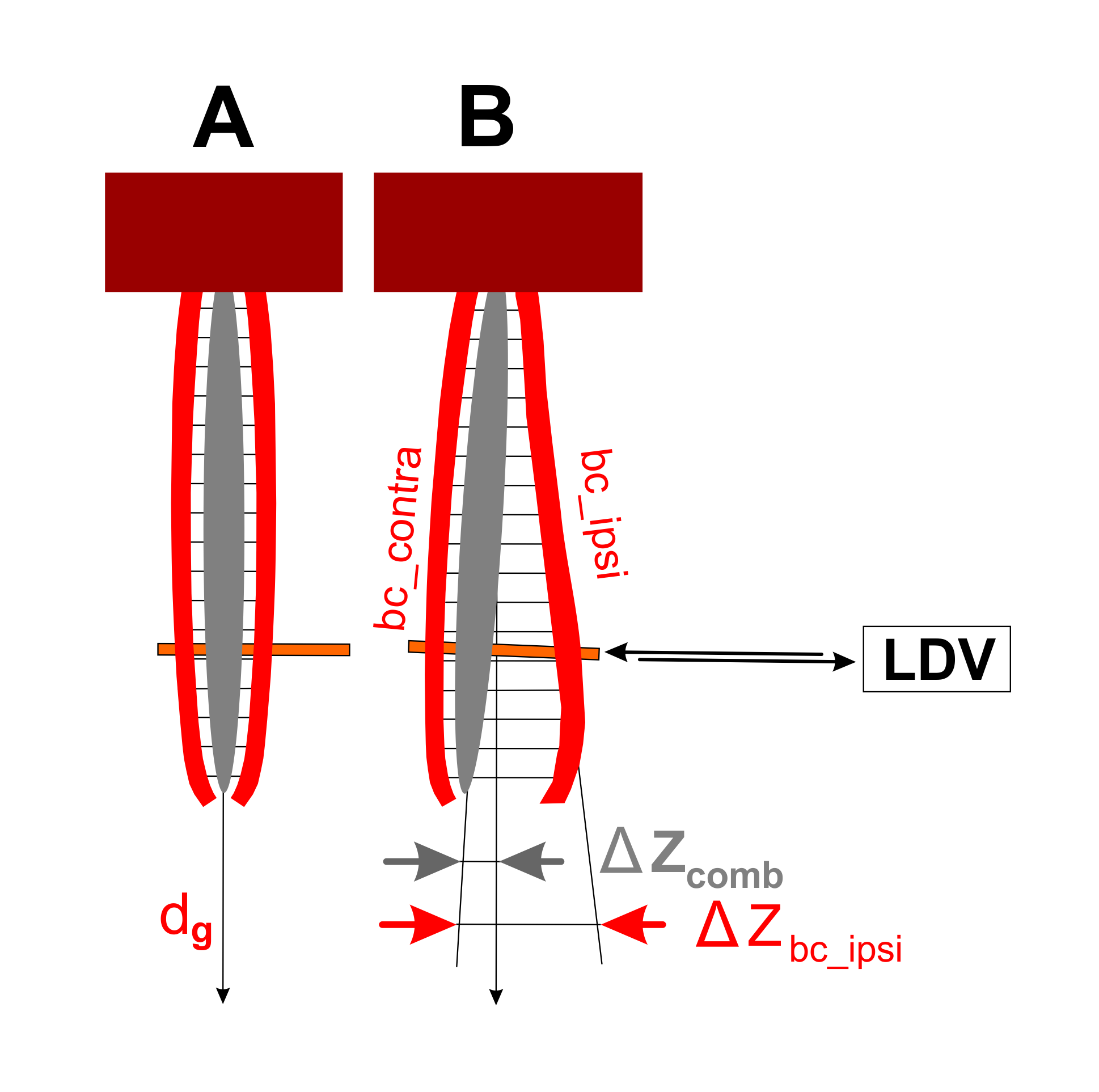

Supplement: S3 Fig — A, quiescent, “non-ventilatory” phase; B, “inhalation” phase. Red areas symbolize the bee curtain, the grey areas the comb; thin black horizontal lines show that the bees of the inner surface of the bee curtain contact the comb with their extremities; longer lines refer to stretched extremities; horizontal orange bar is a wooden rod which was stuck through the comb and the ipsi- and contralateral parts of the bee curtain (bc_ipsi, bc_contra). The LDV ray was reflected on the ipsilateral plane end of the rod. Black vertical arrows give the direction of gravity (dg). The dislocation of the comb (ΔZcomb) from the direction of gravity was measured with LDV and the dislocation of the ipsilateral bee curtain (ΔZbc_ipsi), locally around the convection funnel, was calculated according to Eq 5. (TIF) [file pone.0157882.s003.tif]
